# Latent Sex Differences in CaMKII-nNOS Signaling That Underlie Antidepressant-Like Effects of Yueju-Ganmaidazao Decoction in the Hippocampus

**DOI:** 10.3389/fnbeh.2021.640258

**Published:** 2021-07-06

**Authors:** Ying Yin, Shiyu Qian, Yifan Chen, Yan Sun, Yuqiao Li, Yongfei Yu, Jianqing Li, Zhangjie Wu, Xinlang Yu, Rui Ge, Jia Han, Dongdong Sun, Haoxin Wu, Lanying Liu, Wenda Xue, Wei Wang

**Affiliations:** ^1^Key Laboratory of Integrative Medicine for Brain Diseases, Nanjing University of Chinese Medicine, Nanjing, China; ^2^Department of Psychiatry, Tongde Hospital of Zhejiang Province, Hangzhou, China; ^3^Mental Health Center of Zhejiang Province, Hangzhou, China

**Keywords:** sex difference, YG, nNOS, CaMKII, CREB

## Abstract

Previous studies have demonstrated that Yueju-Ganmaidazao (YG) decoction induces rapid antidepressant-like effects, and the antidepressant response is mostly dependent on the suppression of nitric oxide-cyclic guanosine monophosphate signaling in male mice. This study aimed to investigate the sex difference mediated by calcium/calmodulin-dependent protein kinase II (CaMKII)-neuronal nitric oxide synthase (nNOS) signaling involved in the antidepressant-like effect of YG in mice. We found that the immobility times in the tail suspension test (TST) were found to be decreased after the single injection of YG in male and female mice with the same dosage. Additionally, chronic administration for 4 days of subthreshold dosage of YG and escitalopram (ES) also significantly decreased the immobility time in mice of both sexes. Chronic subthreshold dosage of YG and ES in LPS-treated mice and in chronic unpredictable stress (CUS) mice both decreased the immobility time, which was increased by stress. Meanwhile, in CUS-treated mice, sucrose preference test, forced swimming test, and open field test were applied to further confirm the antidepressant-like effects of YG and ES. Moreover, CUS significantly decreased the expression of nNOS and CaMKII, and both YG and ES could enhance the expression in the hippocampus of female mice, which was opposite to that in male mice, while endothelial nitric oxide synthase expression was not affected by stress or drug treatment neither in male mice nor in female mice. Finally, subthreshold dosage of YG combined with 7-nitroindazole (nNOS inhibitor) induced the antidepressant-like effects both in female and in male mice, while the single use of YG or 7-NI did not display any effect. However, pretreatment with KN-93 (CaMKII inhibitor) only blocked the antidepressant-like effect of high-dosage YG in female mice. Meanwhile, in CUS mice, chronic stress caused NR1 overexpression and inhibited cAMP response element binding protein action, which were both reversed by YG and ES in male and female mice, implying that YG and ES produced the same antidepressant-like effect in mice of both sexes. The study revealed that chronic treatment with a subthreshold dose of YG also produced antidepressant-like effects in female mice, and these effects depended on the regulation of the CaMKII-nNOS signaling pathway.

## Introduction

Depression is one of the most common mental disorders that is associated with high morbidity and mortality, and it affects more than 350 million people in the world (Menard et al., 2016). People suffering from depression always display disrupted mood, altered sleep, and memory dysfunction (Benson et al., 2015). According to the World Health Organization (WHO), depression is the leading cause of disability and will become the second most common disease in the world at 2025. Sex is an important factor in influencing the susceptibility to psychiatric illness (Agarwal et al., 2020). Women are more likely to suffer from anxiety disorders, while men are more likely to suffer from substance-use disorders, suggesting that there are some differences in the mechanisms of depression between the sexes. More importantly, the incidence of depression in females is about two to three times of that in males, and this issue has attracted increasing attention in recent years (Bekker and van Mens-Verhulst, 2007; Essau et al., 2010). Furthermore, evidence suggests that the sex-dependent differences have been observed in the antidepressant-like effects of ketamine, which are believed to modulate the NMDA signaling in the brain, and thus potentially indicates that there is a different underlying pathology between men and women (Carrier and Kabbaj, 2013; Franceschelli et al., 2015). Herein, we designed this study to search for clues underlying the sex differences in depression in the response to antidepressants.

Although a number of antidepressants exist, such as widely used selective serotonin reuptake inhibitors (SSRIs), a remarkable population of patients never attain a sustained remission of their symptoms (Zanos et al., 2016). Fast-acting antidepressants like ketamine may be used to treat depression in patients who have no response to SSRIs and to relieve depressive symptoms quickly (Ballard et al., 2015). Unfortunately, addictive and side effects of ketamine limit its clinical application (Wang et al., 2018). Yueju pill and Ganmaidazao decoction are widely used prescriptions of traditional Chinese medicine. YG (Yueju-Ganmaidazao) decoction has also been demonstrated to induce a rapid and lasting antidepressant-like effect after a single administration in male mice (Zhang H. et al., 2020). However, it remains to be shown whether the chronic or a low dose of YG will still reveal the antidepressant-like effects.

Yueju-Ganmaidazao can produce rapid antidepressant-like effects mostly by reducing the nitric oxide-cyclic guanosine monophosphate (NO-cGMP) pathway in the hippocampus of male mice. Meanwhile, we have found that YG can rapidly enhance the expression of cAMP response element binding protein (CREB) signal in the hippocampus of mice. Furthermore, pharmacological experiments show that blocking the NO-cGMP pathway can reverse the rapid antidepressant-like effect of YG after a single administration, suggesting that NO plays a leading role in mediating the effect (Zhang H. et al., 2020). In addition, escitalopram (ES), one of the commonly used clinical antidepressants, showed a safer profile among antidepressants treated in depression patients (Solmi et al., 2020). ES has been found to depend on the NO pathway to produce antidepressant-like effect (Ludka et al., 2013). In the study, we used ES as a positive control. Our study implied that NO signaling may play an important role in promoting antidepressant-like effect of YG or ES. Our previous work has also demonstrated that the NO concentration can be affected by stress or Yueju pill, which induces depressive or antidepressant-like behavior (Wang et al., 2018). However, most existing research mainly focuses on males, while sex differences in the antidepressant-like effects of drugs remain elusive. Thus, it is essential to focus on whether the NO pathway involved in an antidepressant-like effect is dependent on sex. NO is formed by the NO synthase, endothelial nitric oxide synthase (eNOS) and neuronal nitric oxide synthase (nNOS), and plays an important role in the regulation of antidepressant-like effect in mice. Administration of 7-nitroindazole (7-NI; a nNOS-specific inhibitor) combined with YG can induce antidepressant-like effects in male mice (Zhang H. et al., 2020). It is also shown that nNOS is phosphorylated at Ser847 by calcium/calmodulin-dependent protein kinase II (CaMKII), leading to neuroprotective effects against cerebral ischemia injury (Osuka et al., 2002; Takata et al., 2020). More importantly, nNOS exists in GABAergic inhibitory interneurons where CaMKII co-localizes in the rat hippocampal primary neurons. CaMKII phosphorylates and influences nNOS *via* its specific Ser847 residue (Hayashi et al., 1999; Araki et al., 2020). Meanwhile, like nNOS, CaMKII is a calcium-dependent enzyme (Stein et al., 2020). The CaMKII–nNOS signaling was revealed previously to display neuroprotective effects underlying ischemic preconditioning (Wang et al., 2016), while the action of NO in the adult brain was also demonstrated to be associated with the different expression pattern of CaMKII–nNOS signaling (Packer et al., 2005). Thus, whether the CaMKII–nNOS signaling is participating in the antidepressant-like effect of YG or ES is worth studying clearly.

Based on this, we proposed a hypothesis that the mechanisms involved in sex differentiation of antidepressant-like effects in mice are mediated by the differences in NO changes caused by nNOS, which requires the activation of CaMKII. Here, we aimed to investigate the antidepressant-like response to chronic YG in male and female mice. Then, we determined the expression levels of nNOS as well as eNOS, the potential upstream effector CaMKII and NR1, and examined whether male and female mice exhibit similar or distinct molecular changes in response to stress exposure as well as YG treatment, with a focus on whether the same molecular pathways are influenced similarly across the hippocampus.

## Materials and Methods

### Animals

Male and female ICR mice (aged 7–8 weeks, 20–25 g) were purchased from Shanghai Sippr BK Laboratory Animals Company. Mice were adapted to animal facilities for 1 week before the experiment. Mice were kept in standard laboratory conditions (temperature: 23 ± 2°C; indoor humidity: 50 ± 10%), with a light/dark cycle of 12:12 h, and allowed free access to rodent chow and drinking water. The procedures complied with the Guidelines for the Care and Use of Laboratory Animals and were approved by the Animal Care Committee.

### Preparation of Formula

Yueju-Ganmaidazao decoction was processed and purified as described in our previous work, and the quality control demonstrated that the extract was stable for application (Zhang H. et al., 2020). Briefly, the medicinal plants used to prepare YG decoction were *Cyperus rotundus* L. (CR), *Ligusticum chuanxiong* Hort (LC), *Gardenia jasminoides* Ellis (GJ), *Atractylodes lancea* (Thunb) DC (AL), *Massa fermentata* (MF), *Curabitur triticum* (CB), *Licorice* (LR), and *Fructus Ziziphus Jujuba* (FZJ). The usages of single herbs were CR 100 g: LC 100 g: GJ 100 g: AL 100 g: MF 100 g: CB 125 g: LR 250 g: FZJ 375 g. All medicinal Chinese herbs were purchased from the outpatient department of the School of Medicine. The above materials were soaked with water (1:8 ratio) for 30 min and heated for 1 h, then filtered, and collected. This procedure was repeated twice. The yield of YG extraction was 20% and administrated intragastrically. The dosage of each drug followed the Chinese Pharmacopeia.

### Animal Treatment

#### Chronic Drug Treatment in Normal Mice

Female and male mice were randomly divided into three groups, namely, control group, YG (1 g/kg) group, and ES (10 mg/kg) group, with eight mice in each group. YG and ES groups were injected with drugs for 4 consecutive days, while the control group received the same dose of saline.

### LPS-Induced Procedure

Female and male mice were randomly divided into three groups, namely, LPS-treated control (1 mg/kg) group, LPS + YG (1 g/kg) group, and LPS + ES (10 mg/kg) group, with eight mice in each group. Lipopolysaccharide (LPS, Aladdin, L118716) was prepared in saline (0.9% sodium chloride). LPS (i.p.) was administered once daily, while YG and ES were given by gavage once a day, and the control group was given the same amount of saline; these above administrations were continued for 4 days. Twenty-four hours after the last drug administration, behavioral tests were performed.

### Drugs Interaction Procedure

Female and male mice were pretreated with saline (0.9%) or 7-NI (30 mg/kg, dissolved in 5% Tween 80) or KN-93 (5 mg/kg) for 1 h, and then the mice were treated with the administration of saline, YG (2.5 g/kg, i.g.), or ES (20 mg/kg). After an hour, the tail suspension test (TST) was performed to measure the despair behavior of mice. All drugs were administered i.p. except YG solution.

### Chronic Unpredictable Stress

The CUS experimental protocol was followed as described in Tang et al. (2015). Mice lived alone and received unpredictable stress for 3 weeks. Stress was given in a random, unpredictable order every day: food deprivation 24 h, drink deprivation 24 h, 45°cage tilt for 24 h, cage shaking (high-speed horizontal shaking, 200 rpm) for 40 min, cage wet (200 ml water per cage) for 20 h, overnight illumination and being bound in a 50-ml tube for 6 h. All CUS mice were single-housed until the end of the experiment, and the control mice were normally reared for comparison.

### Tail Suspension Test

The tail of the mouse was taped to the hanging hook, and the tip of the tail was about 1 cm from the tape. Mice were placed in a universal sound-proof behavior box so that they were hung upside down with their heads 20 cm from the bottom of the box. The mice struggled to overcome the abnormal posture, and animal activities were recorded. Any-maze software was used to record the activity of mice for 6 min. The immobility time during the last 4 min was analyzed.

### Forced Swimming Test

The mice were placed in a 10-cm-deep beaker (10cm × 30cm) filled with warm water (25 ± 2°C). Mice were observed for 6 min, and the immobility time during the last 4 min was recorded. When mice floated passively without struggling, they were considered immobile. The immobility time was recorded by using the Any-maze software.

### Sucrose Preference Test

All mice were single-housed and adapted to a sucrose solution (2%) for 72 h according to previous work (Xue et al., 2016). After 18 h of fasting and no drinking, the mice were separated in a single cage and presented with two bottles: one bottle of 2% sucrose solution and one bottle of pure water. The sucrose consumption was observed in 2 h. Sucrose preference was calculated by the formula: sucrose consumption/% = [(sucrose intake) / (sucrose intake + water intake)] × 100%, and was standardized by the weight of each animal.

### Western Blot

Mice were sacrificed, the brains were rapidly removed, and the hippocampus was dissected out and dissolved in a RIPA buffer containing a protease inhibitor and a phosphatase inhibitor. Brain tissues were homogenized, and western blot analyses were carried out. Protein lysates were separated by 8% SDS–PAGE and transferred to a polyvinylidene fluoride (PVDF) membrane. After blocking with 5% BSA for 1 h, the membrane was blocked with primary antibodies for eNOS (Millipore, 1: 1000), nNOS (Millipore, 1: 1000), CaMKII and NR1 (Cell Signaling Technology, 1: 1000), and β-tubulin (Proteintech, 1: 5000) and incubated at 4°C for 12 h, and then the membrane was incubated with the secondary antibody for 1 h at room temperature. The blots were visualized using the SuperSignal West Pico Chemiluminescent Substrate (Thermo Fisher Scientific Inc.). All target proteins were normalized to β-tubulin. All experiments were performed at least three times.

### Statistical Analysis

Multiple comparisons were made using one-way ANOVA or two-way ANOVA followed by Bonferroni *post hoc* tests. All data are indicated as the mean ± SEM and are statistically significant at the 0.05 level.

## Results

### Single Administration of Effective Dosage of YG Displayed the Same Antidepressant-Like Effect in Both Male and Female Mice

**FIGURE 1:**
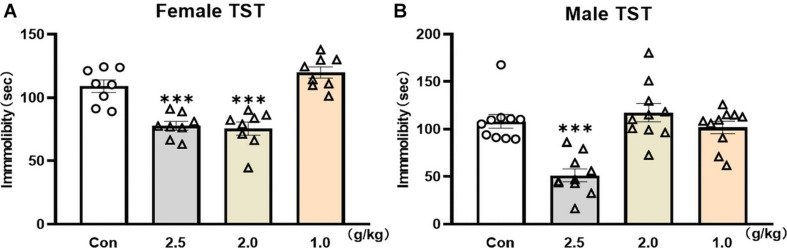
Fast screen of potential rapid antidepressant-like effect of YG in TST of male and female mice. **(A)** Immobility time was measured for the last 4 min during the total 6-min testing time in female mice after different dosages of YG treatments (1.0, 2.0, and 2.5 g/kg). **(B)** Immobility time was measured for the last 4 min during the total 6-min testing time in male mice after different dosages of YG treatments (1.0, 2.0, and 2.5 g/kg). Independent mouse was used to test the behaviors at each time point. ^∗∗∗^*p* < 0.001, compared with the control group, one-way ANOVA, *n* = 8–10/group.

The TST was used to screen rapid antidepressant-like effects of YG in female and male mice. Twenty-four hours after a single administration of YG, the immobility time was significantly reduced in female mice [Figure 1A, one-way ANOVA, *F*(3,28) = 23.88, *p* < 0.0001] both at the dosage of 2.5 g/kg (*p* < 0.001) and at the dosage of 2.0 g/kg (*p* < 0.001), and 2.0 g/kg was equivalent to the human clinical dose. Meanwhile, in male mice, the immobility time [Figure 1B, one-way ANOVA, *F*(3,36) = 15.04, *p* < 0.0001] was significantly decreased only at the dosage of 2.5 g/kg (*p* < 0.001). The results showed that YG produced the same antidepressant-like effect in female and male mice, but the effective dosage range was wider in female mice.

### Subthreshold Dosage of YG Could Also Induce Antidepressant-Like Effect in LPS-Induced Model Both in Male and in Female Mice

**FIGURE 2:**
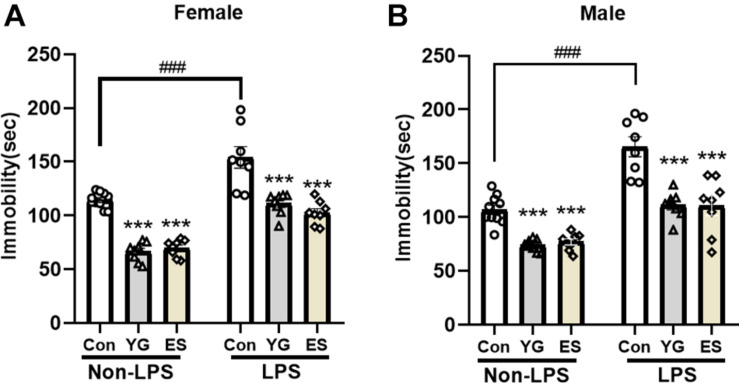
Chronic YG (1 g/kg) and ES (10 mg/kg) displayed significant effects on immobility times in the TST both in **(A)** female and in **(B)** male mice as well as in non-LPS treatment or LPS treatment. ***p < 0.001, compared with the control group in non-LPS or LPS treatment, one-way ANOVA, *n* = 8–10/group. The interaction between non-LPS and LPS treatment was analyzed by two-way ANOVA. ^###^*p* < 0.001 compared to non-LPS control group.

Previous studies have focused on the role of the rapid antidepressant-like action of YG, but the chronic treatment with the formula is more common clinically. The subchronic mice with LPS-injection for 4 consecutive days were used to evaluate the antidepressant-like effect of YG and ES. LPS is a well-known proinflammatory drug and is widely employed to induce the depressive-like behavior (Ali et al., 2020); 1 g/kg of YG (*p* < 0.001) and 10 mg/kg of ES (*p* < 0.001) both produced antidepressant-like effects in female mice [Figure 2A, left hind paw: one-way ANOVA, *F*(2,23) = 103.9, *p* < 0.0001] after chronic injections. Meanwhile, immobility times of male mice during the TST [Figure 2B, left hind paw: one-way ANOVA, *F*(2,23) = 30.24, *p* < 0.0001] were significantly decreased in YG-treated group (*p* < 0.001) and ES-treated group (*p* < 0.001).

After chronic treatment with YG (*p* < 0.001) or ES (*p* < 0.001) in LPS-treated mice, the immobility time was significantly decreased compared to that of the control group [Figure 2A, right hind paw: one-way ANOVA, *F*(2,21) = 17.93, *p* < 0.0001]. In male mice [Figure 2B, right hind paw: one-way ANOVA, *F*(2,21) = 15.73, *p* < 0.0001], chronic treatment of YG (*p* < 0.001) and ES (*p* < 0.001) both induced significant antidepressant-like effects. After treating with LPS, the immobility time was significantly increased both in female (*p* < 0.001) and in male (*p* < 0.001) mice. This effect was not affected by sex [two-way ANOVA, female interaction: *F*(2,44) = 0.7460, *p* = 0.4802, male interaction: *F*(2,44) = 2.686, *p* = 0.0793]. Collectively, the antidepressant-like effects of chronic YG and ES showed no significant sex differences.

### Chronic Administration of Subthreshold Dose YG Could Also Induce Antidepressant-Like Effect in CUS Female and Male Mice

**FIGURE 3:**
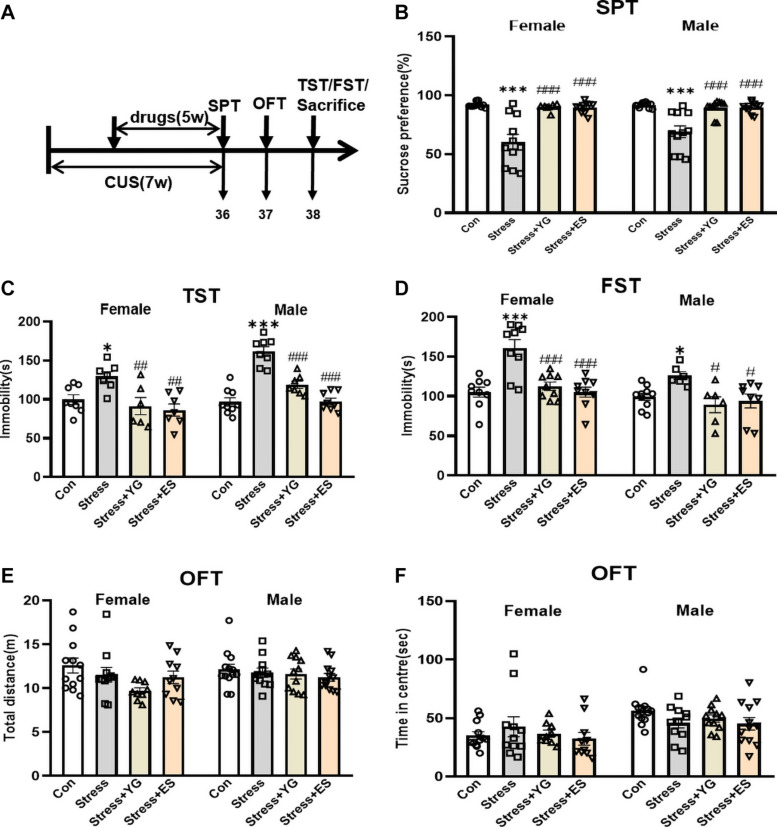
Antidepressant-like behaviors following chronic YG or ES in CUS mice. **(A)** Mice were exposed to chronic unpredictable stress (CUS) and received treatment of YG or ES. Control (Con) mice were not exposed to stress but received saline treatment. Behaviors were tested after the last drug administration. **(B)** Mice were tested in the SPT after the last drug administration. **(C)** Mice were tested in the TST in 2 days after the last drug administration. **(D)** Mice were tested in the FST in 2 days after the last drug administration. Mice were tested in the OFT in 1 day after the last drug administration: **(E)** total distance and **(F)** time in the center. ^∗^*p* < 0.05, ^∗∗∗^*p* < 0.001, compared with the control group; ^#^*p* < 0.05, ^##^*p* < 0.01, ^###^*p* < 0.001, compared with the CUS group. One-way ANOVA, *n* = 6–13/group.

Both female and male mice received CUS for 2 weeks. Then, mice were given either saline or YG (1 g/kg) or ES (10 mg/kg) for 5 weeks after the establishment of CUS model. After the last administration of saline or drug, the immobility time in the TST and in the forced swimming test (FST) was measured in both sexes, and the sucrose preference test (SPT) was used to evaluate anhedonia-like stage of the mice (Figure 3A). The results showed that, compared with the saline group, the preferences for sucrose solution of female mice [Figure 3B, one-way ANOVA, *F*(3,35) = 17.99, *p* < 0.0001] and male mice [Figure 3B, one-way ANOVA, *F*(3,39) = 14.08, *p* < 0.0001] were significantly reduced (female: *p* < 0.001, male: *p* < 0.001). After 5 weeks of treatment, both YG (*p* < 0.001) and ES (*p* < 0.001) could rescue the anhedonia phenomena induced by CUS. The total solution (containing sucrose or not) was not affected by stress or drugs treatment (Supplementary Figure 1). In the TST, the immobility time was significantly increased after stress [Figure 3C, one-way ANOVA, female: *F*(3,24) = 6.090, *p* < 0.01, *p* < 0.05 compared to the control group; male: *F*(3,28) = 34.00, *p* < 0.0001, *p* < 0.001 compared to Con group], while YG (female: *p* < 0.01, male: *p* < 0.001) and ES (female: *p* < 0.01, male: *p* < 0.001) significantly decreased the immobility time compared to the CUS group. In the FST, the CUS group displayed higher immobility time, while YG group (female: *p* < 0.001, male: *p* < 0.05) and ES group (female: *p* < 0.001, male: *p* < 0.05) showed lower immobility times in both male and female mice [Figure 3D, one-way ANOVA, female: *F*(3,32) = 13.19, *p* < 0.0001; male: *F*(3,26) = 4.355, *p* < 0.05]. Meanwhile, the locomotor activity was not affected by stress or drugs in total distance [Figure 3E, one-way ANOVA, female: *F*(3,38) = 2.399, *p* = 0.0830; male: *F*(3,43) = 0.7807, *p* = 0.5112] or in traveling time in center area [Figure 3F, one-way ANOVA, female: *F*(3,38) = 0.5968, *p* = 0.6210; male: *F*(3,43) = 1.656, *p* = 0.1907] in the open field test (OFT). We found no significant sex differences after stress or drug treatments [two-way ANOVA, Figure 3B: *F*(3,74) = 0.9566, *p* = 0.4179; Figure 3C: *F*(3,52) = 3.211, *p* = 0.0304; Figure 3D: *F*(3,58) = 1.271, *p* = 0.2928; Figure 3E: *F*(3,81) = 0.5919, *p* = 0.6221; Figure 3F: *F*(3,81) = 1.211, *p* = 0.3110].

### Subthreshold Dosage of YG Combined With 7-NI Could Induce an Antidepressant-Like Effect on Female and Male Mice

**FIGURE 4:**
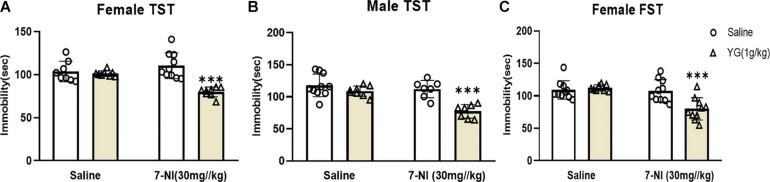
Antidepressant-like effects of YG following nNOS intervention. The effect of 7-NI (30 mg/kg, i.p.) pretreatment on the antidepressant response of YG (1 g/kg, i.g.) in the TST of **(A)** female and **(B)** male mice. Meanwhile, **(C)** FST was also used to evaluate the interaction between YG and 7-NI. ^∗∗∗^*p* < 0.001, compared with Control + Saline. Two-way ANOVA, *n* = 7–10/group.

The role of nNOS in YG’s antidepressant-like action was assessed by using 7-NI, a nNOS inhibitor. 7-NI (30 mg/kg) inhibited the antidepressant-like effect of YG (1 g/kg) in female mice [two-way ANOVA, main effect of treatment, *F*(1,30) = 23.88, *p* < 0.001; pretreatment, *F*(1,30) = 4.071, *p* < 0.05; treatment × pretreatment interaction, *F*(1,30) = 20.38, *p* < 0.001, Figure 4A]. 7-NI (30 mg/kg) also inhibited the antidepressant-like effect of YG (1 g/kg) in male mice [two-way ANOVA, the main effect of treatment, *F*(1,28) = 14.91, *p* < 0.001; pretreatment, *F*(1,28) = 20.65, *p* < 0.001; treatment × pretreatment interaction, *F*(1,28) = 7.205, *p* < 0.05, Figure 4B]. Results displayed that YG combined with 7-NI could produce antidepressant-like effects both in female and in male mice (Zhang H. et al., 2020). Furthermore, in the FST, 7-NI (30 mg/kg) inhibited the antidepressant-like effect of YG (1 g/kg) in female mice [two-way ANOVA, the main effect of treatment, *F*(1,34) = 10.46, *p* < 0.01; pretreatment, *F*(1,34) = 12.25, *p* < 0.01; treatment × pretreatment interaction, *F*(1,34) = 6.625, *p* < 0.05, Figure 4C].

### nNOS and CaMKII Showed Different Expression Patterns After Stress or Drug Treatment in Male and Female Mice

**FIGURE 5:**
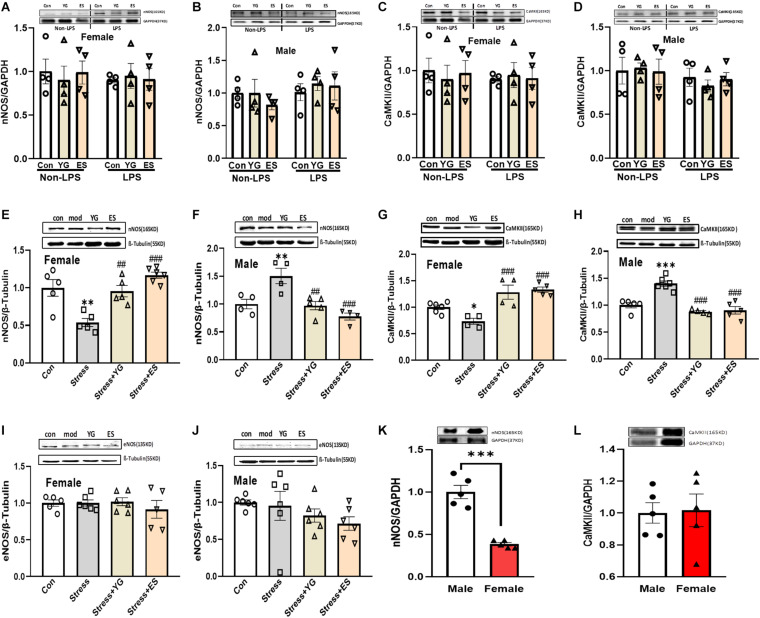
CaMKII, nNOS, and eNOS expression in the hippocampus after chronic administration of stress, YG and ES in female and male mice. **(A)** nNOS expression in female mice in non-LPS or LPS treatment. **(B)** nNOS expression in male mice in non-LPS or LPS treatment. **(C)** CaMKII expression in female mice in non-LPS or LPS treatment. **(D)** CaMKII expression in male mice in non-LPS or LPS treatment. **(E)** nNOS expression in female mice after CUS or drug treatment. **(F)** nNOS expression in male mice after CUS or drug treatment. **(G)** CaMKII expression in female mice after CUS or drug treatment. **(H)** CaMKII expression in male mice after CUS or drug treatment. **(I)** eNOS expression in female mice after CUS or drug treatment. **(J)** eNOS expression in male mice after CUS or drug treatment. **(K)** nNOS expression in normal female and male mice. **(L)** CaMKII expression in normal female and male mice. ^∗^*p* < 0.05, ^∗∗^*p* < 0.01, ^∗∗∗^*p* < 0.001 compared with the control group; ^##^*p* < 0.01, ^###^*p* < 0.001, compared with the CUS group, one-way ANOVA or two-way ANOVA, *n* = 4–6/group.

Our previous work found that the antidepressant-like effect of YG was dependent on NO-cGMP pathway. In this study, we investigated whether the NOS expression was affected by chronic YG or ES in mice of different sexes. After chronic treatment with YG or ES in mice, neither in female mice [Figure 5A, left hind paw, one-way ANOVA, *F*(2,9) = 0.1409, *p* = 0.8705] nor in male mice [Figure 5B, left hind paw, one-way ANOVA, *F*(2,9) = 0.5718, *p* = 0.5838], nNOS expression was not affected by the drugs. In LPS-treated mice, we also found that the expression of nNOS was not affected by YG or ES in female mice [Figure 5A, right hind paw, one-way ANOVA, *F*(2,9) = 0.05090, *p* = 0.9506] or in male mice [Figure 5B, right hind paw, one-way ANOVA, *F*(2,9) = 0.1868, *p* = 0.8327]. Neither LPS treatment nor sex showed differences in nNOS expression by using two-way ANOVA [female interaction: *F*(2,18) = 0.1887, *p* = 0.8297; male interaction: *F*(2,18) = 0.4441, *p* = 0.6483]. Meanwhile, CaMKII expression was similar to the nNOS expression in the hippocampus [Figure 5C, left hind paw, one-way ANOVA, *F*(2,9) = 0.1161, *p* = 0.8917; right hind paw, one-way ANOVA, *F*(2,9) = 0.03030, *p* = 0.9703; Figure 5D, left hind paw, one-way ANOVA, *F*(2,9) = 0.05090, *p* = 0.9506; right hind paw, one-way ANOVA, *F*(2,9) = 0.3785, *p* = 0.6953; two-way ANOVA, female interaction: *F*(2,18) = 0.1660, *p* = 0.8483; male interaction: *F*(2,18) = 0.2262, *p* = 0.7998]. Furthermore, after CUS exposure, the expression of nNOS [Figure 5E, one-way ANOVA, *F*(3,17) = 13.84, *p* < 0.0001] was significantly decreased (*p* < 0.01), which was reversed by YG (*p* < 0.01) and ES (*p* < 0.001) treatment in female mice. However, in male mice [Figure 5F, one-way ANOVA, *F*(3,13) = 10.60, *p* < 0.001], chronic stress (*p* < 0.01) significantly increased nNOS expression, while YG (*p* < 0.01) and ES (*p* < 0.001) decreased the overexpression of nNOS. Meanwhile, CaMKII protein expression was found to be similar to nNOS in female and male mice [Figure 5G, one-way ANOVA, *F*(3,15) = 14.21, *p* < 0.0001; Figure 5H, one-way ANOVA, *F*(3,16) = 23.13, *p* < 0.0001]. We also investigated whether the eNOS participated in the antidepressant-like effect of YG and ES. The results showed that eNOS was not affected by stress or drugs neither in female [Figure 5I, one-way ANOVA, *F*(3,18) = 0.4326, *p* = 0.7322] nor in male mice [Figure 5J, one-way ANOVA, *F*(3,20) = 1.196, *p* = 0.3367]. These results suggested that although female and male mice exhibited a consistent phenotype under stress or drug action, their potential molecular mechanisms might be different and might be associated with CaMKII-nNOS signaling pathways. In the normal mice, the protein expression levels of nNOS and CaMKII at the basal level were also measured, and the results showed that only nNOS expression in female mice was lower than that in male mice (Figure 5K, *t*-test, *p* < 0.001; Figure 5L, *t*-test, *p* = 0.8938).

### KN-93 Only Block the Antidepressant-Like Effect of YG and ES in Female Mice

**FIGURE 6:**
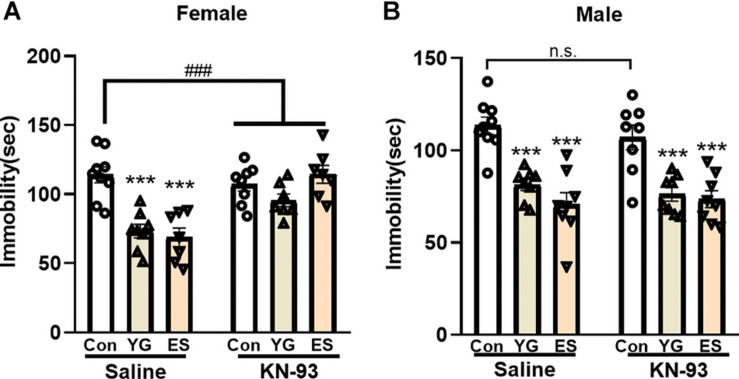
The effect of pretreatment with saline or KN-93 on single YG (2.5g/kg) or ES (20mg/kg) administration during the TST paradigm in **(A)** female and **(B)** male mice. *** *p* < 0.001, compared with the control group of saline pretreatment, one-way ANOVA, *n* = 7-9/group. The interaction between saline and KN-93 pretreatment was analyzed by two-way ANOVA. ^###^*p* < 0.001 compared to non-LPS control group.

To explore whether the antidepressant-like effect of YG or ES depends on CaMKII expression, KN-93 (a specific CaMKII inhibitor) was used to pretreat the mice before YG or ES. In the normal mice, high dosage of YG (2.5 g/kg, *p* < 0.001) and ES (20 mg/kg, *p* < 0.001) significantly decreased the immobility time after single injection both in female [Figure 6A, left hind paw, one-way ANOVA, *F*(2,21) = 18.90, *p* < 0.0001] and male mice [Figure 6B, left hind paw, one-way ANOVA, *F*(2,22) = 21.62, *p* < 0.0001]. However, after KN-93 pretreatment for 1 h before YG or ES administration, the antidepressant-like effect induced by YG or ES was blocked by KN-93 in female mice [Figure 6A, right hind paw, two-way ANOVA, interaction: *F*(2,40) = 10.88, *p* = 0.0002]. Meanwhile, in male mice, KN-93 [Figure 6B, right hind paw, one-way ANOVA, *F*(2,21) = 13.05, *p* < 0.0001] could not blunt the antidepressant-like effect of YG (*p* < 0.001) or ES (*p* < 0.001).

### YG and ES Could Regulate the NR1-CREB Signaling Pathway to Display the Same Antidepressant-Like Behavior Phenotype in Female and Male

**FIGURE 7:**
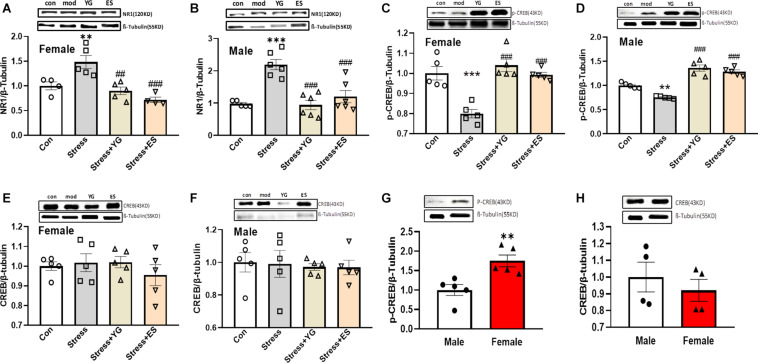
NR1, pCREB, and CREB expression in the hippocampus after chronic subthreshold dosage administration of YG and ES in CUS mice. **(A)** NR1 expression in female mice after CUS or drug treatment. **(B)** NR1 expression in male mice after CUS or drug treatment. **(C)** pCREB expression in female mice after CUS or drug treatment. **(D)** pCREB expression in male mice after CUS or drug treatment. **(E)** CREB expression in female mice after CUS or drug treatment. **(F)** CREB expression in male mice after CUS or drug treatment. **(G)** pCREB expression in normal female and male mice. **(H)** CREB expression in normal female and male mice. ^∗∗^*p* < 0.01, ^∗∗∗^*p* < 0.001, compared with the control group; ^##^*p* < 0.01, ^###^*p* < 0.001, compared with the CUS group, one-way ANOVA, *n* = 4–6/group.

To find the potential mechanism underlying the antidepressant-like effect in female and male mice after YG and ES treatments, we first detected NR1 protein expression in mice. After administration with a subthreshold dose of YG [Figure 7A, one-way ANOVA, *F*(3,14) = 12.15, *p* < 0.001], NR1 expression (*p* < 0.01) was significantly reduced compared to the CUS group, which was significantly increased compared to the control group, and this result was similar to the result of ES administration (*p* < 0.001) in female mice. In male mice, NR1 protein expression showed a similar decreasing tendency [Figure 7B, one-way ANOVA, *F*(3,19) = 15.50, *p* < 0.0001]. Meanwhile, we further measured the phosphorylation CREB and total CREB expressions, which were considered as factors affecting by NR1 signaling. After the CUS procedure, pCREB expression was significantly decreased (*p* < 0.001), and YG (*p* < 0.001) and ES (*p* < 0.001) up-regulated pCREB expression in female mice [Figure 7C, one-way ANOVA, *F*(3,16) = 17.41, *p* < 0.0001]. In male mice, pCREB [Figure 7D, one-way ANOVA, *F*(3,16) = 15.31, *p* < 0.0001] displayed the same trend as in female mice. Meanwhile, total CREB was not affected by stress or drug treatment in female mice [Figure 7E, one-way ANOVA, *F*(3,16) = 0.5959, *p* = 0.6268] or male mice [Figure 7F, one-way ANOVA, *F*(3,16) = 0.07369, *p* = 0.9732]. Meanwhile, the ratio of pCREB/CREB was significantly decreased compared to control group both in female mice [one-way ANOVA, *F*(3,15) = 9.325, *p* = 0.001] and in male mice [one-way ANOVA, *F*(3,15) = 9.544, *p* < 0.001], and YG and ES could rescue the deficit. Also, we measured the baseline of pCREB and total CREB in mice, and the results showed that pCREB (Figure 7G, *p* < 0.01) displayed higher expression in female mice, while the total CREB (Figure 7H) showed no difference in mice of both sexes.

## Discussion

In the present study, we have characterized the antidepressant-like response of YG in male and female mice as well as the potential neurobiological mechanisms in both sexes. We found that: (1) single treatment with a high dose of YG could produce antidepressant-like effects both in female and in male mice, while the effective dosage in female mice was wider than that in male mice; (2) chronic administration of a subthreshold dose of YG or ES could produce antidepressant-like effects both in female and in male mice as well as in normal or LPS-treated mice; (3) a subthreshold dose of YG and ES could reverse the depressive behavior induced by chronic mild stress without sex differences; (4) nNOS expression showed different change patterns in female and male mice after stress or drug treatment, and 7-NI (nNOS inhibitor) combined with a subthreshold dose of YG could effectively induce antidepressant-like effects both in female and in male mice, indicating that YG revealed nNOS activity-dependent antidepressant-like effects in both sexes; (5) CaMKII expression also displayed the different change pattern in female and male mice after stress or drug treatment; however, KN-93 (CaMKII inhibitor) only blocked the antidepressant-like effect of YG or ES in female mice, implying that YG and ES produced antidepressant-like effects, which mostly depended on the CaMKII-nNOS pathway in female mice; (6) YG and ES could repair the abnormal expression of NR1-CREB signaling caused by stress and thus formed consistent antidepressant-like phenotypes in mice of both sexes.

Our group previously demonstrated that the ethanol extract of Yueju pill induced a rapid and long-lasting antidepressant-like effect in male mice (Xue et al., 2013, 2016; Tang et al., 2015). However, higher dose of ethanol extract of Yueju pill possibly caused side effects in patients compared to the one of the water extracts. A meta-analysis of GM (Ganmai Dazao) decoction in patient for depression suggested that GM showed an antidepressant action without side effects. GM in combination with regular antidepressants significantly reduced the side effects and enhanced the antidepressant efficacies (Yeung et al., 2014). Another study also revealed that modified GM induced an equivalent efficacy to melitracen-flupentixol in climacteric depression (Ma et al., 2014). Subchronic injection of GM reduced CUS-induced depressive-like behavior in rats, and this was associated with a reduction in glutamate levels and increased the expression of NR2A and NR2B in the hippocampus (Lou et al., 2010; El-Alfy et al., 2012). Thus, the water extraction of Yueju pill combined with GM, which has been widely used in clinical traditional Chinese medicine treatment without significant side effects, showed significant antidepressant-like effects after a single injection in male mice. In the present study, we further illustrated that YG displayed antidepressant-like effects in female mice with the same dosage as in male mice. Lower dose of drugs means less risk of side effects, and we investigated whether a low dose of YG induced antidepressant-like effects. Interestingly, in the normal mice, LPS-induced mice, and CUS-treated mice, a low dose of YG as well as the positive control ES all produced the antidepressant-like effects without sex differences. It is implied that lower doses of drugs can safely be used to treat depression such as YG and ES. Previous research showed that the therapeutic effects of a low dose of antidepressants merely disappeared, instead of becoming aversive or toxic (Fitzgerald et al., 2020). However, chronic treatment with antidepressants was more popular. Although a high dose of YG was not found to have side effects, the lower dose of drug means a less risk of side effects. In our study, chronic treatment with a low dose of YG was demonstrated to have the same effect as a high dose of YG.

To find the possible mechanisms of the antidepressant-like effects of YG, we investigated nNOS expression, which was reported to show sex-dependent effects after stress. First, mice were treated with a single treatment of YG, and the effective doses of YG in male and female mice were determined. The subthreshold dose of YG (1 g/kg) combined with 7-NI induced antidepressant-like effects in female and male mice. These results indicated that there were sex differences in nNOS expression after stress or drug treatment. Moreover, the eNOS expression in female and male mice was not affected by stress or drug treatment. These results suggested that nNOS was the key to the antidepressant-like effect of YG and led to the sex differences, rather than eNOS. This is consistent with Hu et al.’s (2012) statement that there is a significant sex difference in NO levels catalyzed by nNOS in the hippocampus after stress exposure. Meanwhile, in the normal mice, nNOS expression was lower in female mice than that in male mice, and the result might supply a clue for the susceptibility to depression in female mice.

To investigate how nNOS is involved in the antidepressant-like effects of YG, we also used ES as the positive control. We measured the expression of nNOS and eNOS in the hippocampus of mice, and the results showed that the depressive-like behaviors in both sexes were significantly improved when compared with the CUS group, and in male mice, YG and ES decreased the expression of nNOS, but the eNOS expression did not change significantly. Previous studies also reported that conventional antidepressants like ES inhibited nNOS activity in male mice or rats (Angulo et al., 2001; Ulak et al., 2008; Krass et al., 2010). However, there are few reports regarding female rodents. In female mice, YG and ES increased the expression of nNOS, but did not change the expression of eNOS. Both YG and ES have the same effects on the depressive-like behavior. This evidence indicates that nNOS is an important factor contributing to the antidepressant-like effects of YG and ES treatment in depressive male and female mice.

Furthermore, we explored the NMDA-CaMKII signal pathway which is upstream of nNOS. As a downstream signal factor of the NMDAR subunit, CaMKII is an extremely abundant protein kinase in brain tissue that participates in a variety of signaling cascade reactions and is an important mediation center for the regulation of learning and memory (Zhang C. et al., 2020). CaMKII has been shown to reposition within the NMDAR complex in response to non-ionotropic NMDAR signaling (Aow et al., 2015; Stein et al., 2020). Our results showed that the expression of CaMKII in the hippocampus of male mice decreased, while in female mice the protein expression increased compared to the control group. The expression of nNOS revealed a similar trend in mice of both sexes after stress. Meanwhile, after YG or ES treatment, both CaMKII and nNOS expression was significantly adjusted to the baseline level. We speculated that the differences between male mice and female mice were mostly dependent on nNOS differential expression, which was activated by CaMKII. CaMKII is known to activate and translocate from the cytoplasm to the synaptic density where nNOS is predominantly located (Araki et al., 2020). Some reported that phosphorylation of nNOS at Ser847 by CaMKII attenuated the NO synthesis activity of nNOS *in vitro* and in cells (Hayashi et al., 1999; Komeima et al., 2000). CaMKII phosphorylates at Ser741 could also lead to a reduction of nNOS activity by blocking the binding of Ca^2+^/CaM (Song et al., 2004; Takata et al., 2020). nNOS is a calcium-dependent enzyme, and we find that nNOS requires downstream of CaMKII signaling in both sexes, and the results are similar to those reported by Stein et al. (2015). Furthermore, the antidepressant-like effects of a high dose of YG and ES were prevented by the presence of KN-93 (CaMKII inhibitor) in female mice but not in male mice, while KN-93 alone showed no effect on the immobility time in mice. After corticosterone exposure, the increase in AMPAR surface trafficking can be pharmacologically modulated by tianeptine in a CaMKII-dependent mechanism (Zhang et al., 2013). In this study, there were no significant differences in CaMKII expression between male and female mice. A previous study reported that CaMKII activity was required for the expression and not initiation of E2-induced synaptic potentiation in female mice (Jain et al., 2019). Thus, this study clearly emphasized that the antidepressant-like effects of YG and ES mostly depended on the CaMKII-nNOS pathway in female mice.

The molecular mechanisms of the antidepressant-like effects of YG and ES are different between the sexes, but the antidepressant-like phenotypes in both sexes are consistent. The expression of NR1, which is one of the important upstream regulators of nNOS, was decreased in the hippocampus of male and female mice after YG and ES treatment when compared to the CUS mice. Additionally, our results showed that, compared to the control group, the expressions of phosphorylation CREB were both decreased in the hippocampus of mice after stress and were reversed by YG and ES without the sex differences. The pathology of depression was caused by the decreases in neuroplasticity in emotion-related brain regions (Hu et al., 2012), and the stress decreased the expression of CREB in the hippocampus of mice. Inhibition of CaMKIIβ-ERK1/2-CREB signaling mediates the chronic ketamine use-associated cognitive impairments by restraining synaptic signaling (Luo et al., 2020). Supplementation of curcumin increases the ratio of pCREB to CREB and corrects the depressive-like behaviors successfully in CUS-treated rats (Liao et al., 2020). CREB signaling, which is inhibited by over-activated GluN2B, participates in the antidepressant-like effects of ketamine, and extrasynaptic CaMKIIα is also involved in the CREB signaling. Studies have indicated that CREB plays an important role in the antidepressant-like effect in rodents. We also found that pCREB expression revealed a basic difference between female and male mice. These results indicate that NR1-CREB displays similar patterns without sex differences after stress or antidepressants, but the potential mechanism might be different.

## Conclusion

In summary, we first confirmed that YG decoction induced stable antidepressant-like effects both in male and in female mice by using a subthreshold effective dosage in different CUS mice. We speculated that the antidepressant-like effects of YG worked through the nNOS pathway, which also had the function of improving downstream synaptic plasticity, and the changes of nNOS expression showed significant sex differences. Finally, we have illustrated that nNOS is modulated by CaMKII but not NR1 in mice after chronic treatment with YG or ES. The CaMKII-nNOS signaling pathway could enhance CREB activity to induce the same antidepressant-like effects in female mice. Furthermore, we want to investigate which subtype of CaMKII (α, β, γ, and δ) plays a dominant role in the mechanism underlying the antidepressant-like effects of YG or ES as well as a potential specific role of estrogen in regulating CaMKII expression in female mice.

## Data Availability Statement

The original contributions presented in the study are included in the article/Supplementary Material, further inquiries can be directed to the corresponding author/s.

## Ethics Statement

The animal study was reviewed and approved by the Nanjing University of Chinese Medicine.

## Author Contributions

WW and WX conceived and designed the experiments. SQ, YY, YC, YS, YL, YFY, JL, ZW, XY, RG, and JH performed the experiments. SQ, YY, YC, WW, and WX analyzed the data. SQ, YY, YC, DS, HW, WW, and WX contributed to the writing of the manuscript. Special thanks to Professor LL for revising this manuscript. All authors contributed to the article and approved the submitted version.

## Conflict of Interest

The authors declare that the research was conducted in the absence of any commercial or financial relationships that could be construed as a potential conflict of interest.
