# Supplementary material for: Latent Sex Differences in CaMKII-nNOS Signaling That Underlie Antidepressant-Like Effects of Yueju-Ganmaidazao Decoction in the Hippocampus
Source: Front Behav Neurosci. 2021 Jul 6;15:640258. doi: 10.3389/fnbeh.2021.640258 (PMC8290083; doi:10.3389/fnbeh.2021.640258)
Supplement: Supplementary file 1 [file Data_Sheet_1.PDF]

Supplementary information

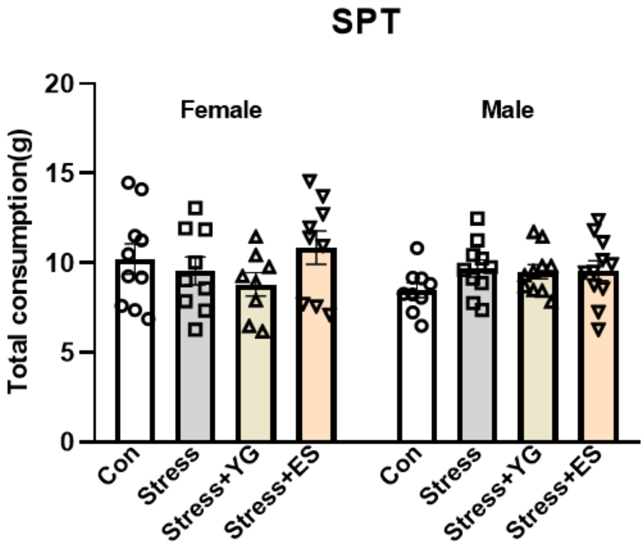

Fig S1 Female and male mice were measured in sucrose preference test after last drugs administration. Total consumptions of water contained sucrose or not were analyzed. n=8-11/group.

nNOS	Female	nNOS-1<br>Tubulin-1<br>nNOS-2<br>Tubulin-2	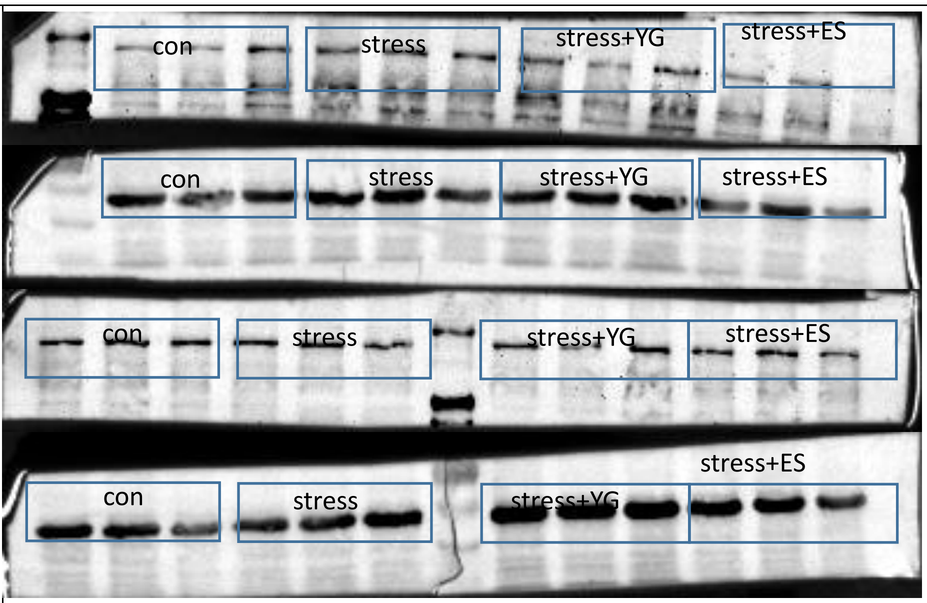
	Male	nNOS-1<br>Tubulin-1<br>nNOS-2<br>Tubulin-2	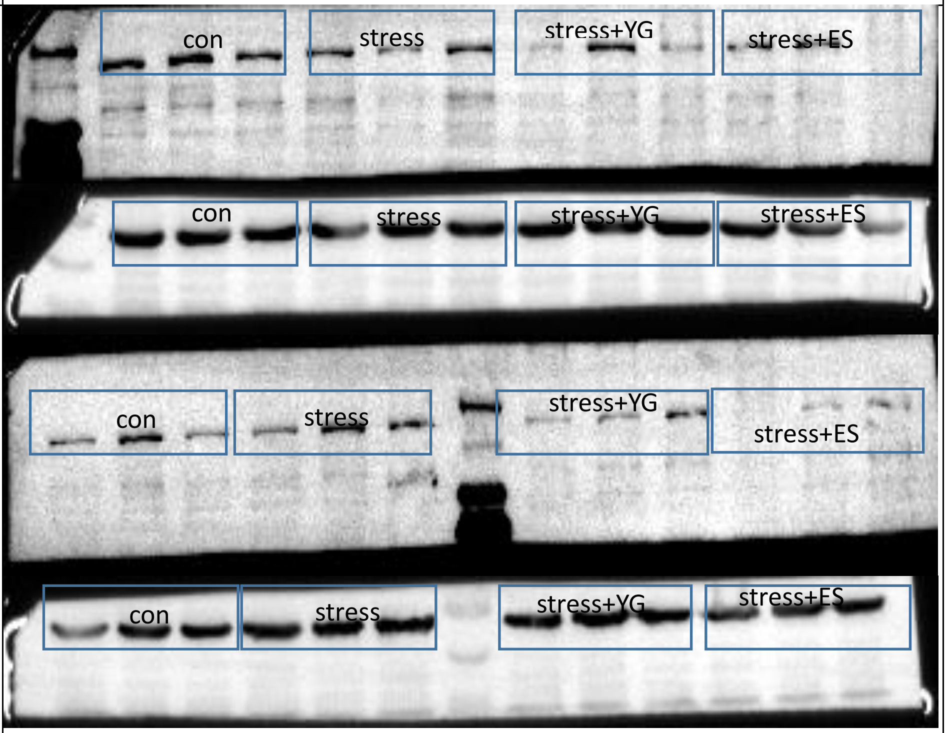
eNOs	Female	eNOS-1<br>Tubulin-1<br>eNOS-2<br>Tubulin-2	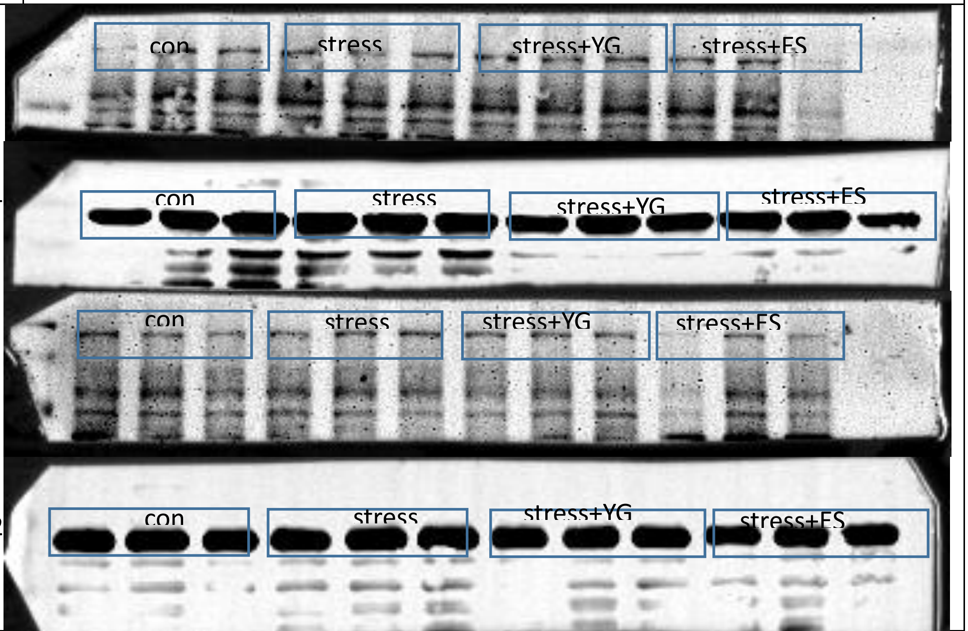

	Male	nNOS-1<br>Tubulin-1<br>nNOS-2<br>Tubulin-2	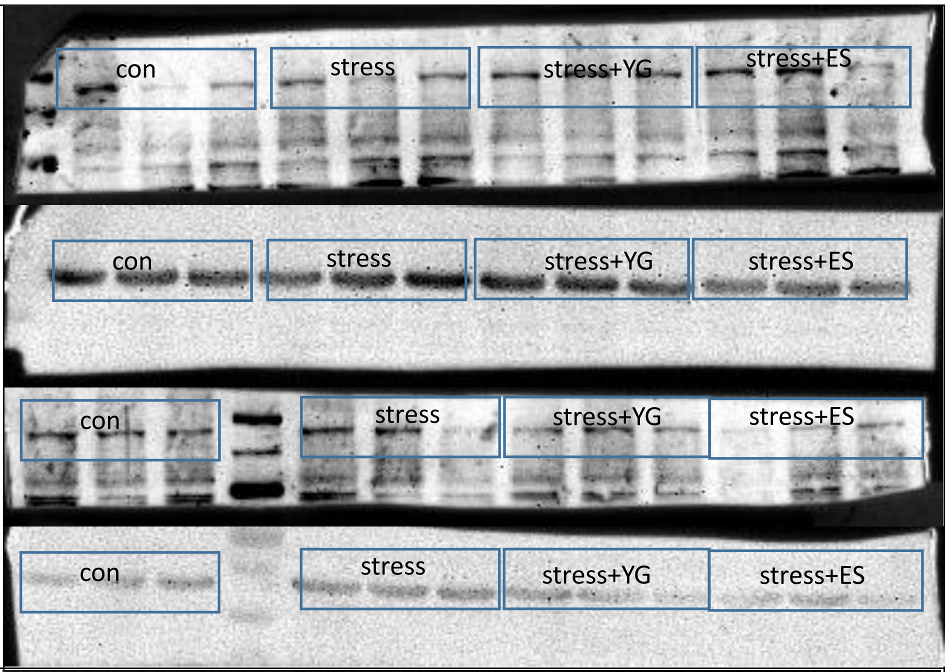
NR1	Female	NR1-1<br>Tubulin-1<br>NR1-2<br>Tubulin-2	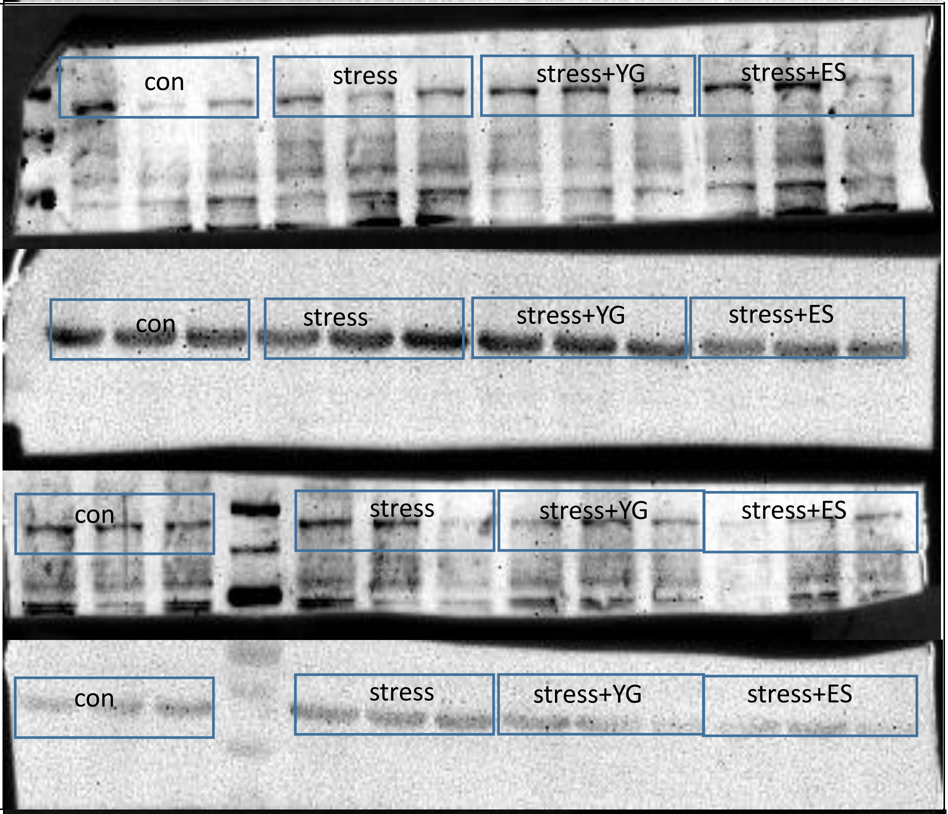
	Male	NR1-1<br>Tubulin-1<br>NR1-2<br>Tubulin-2	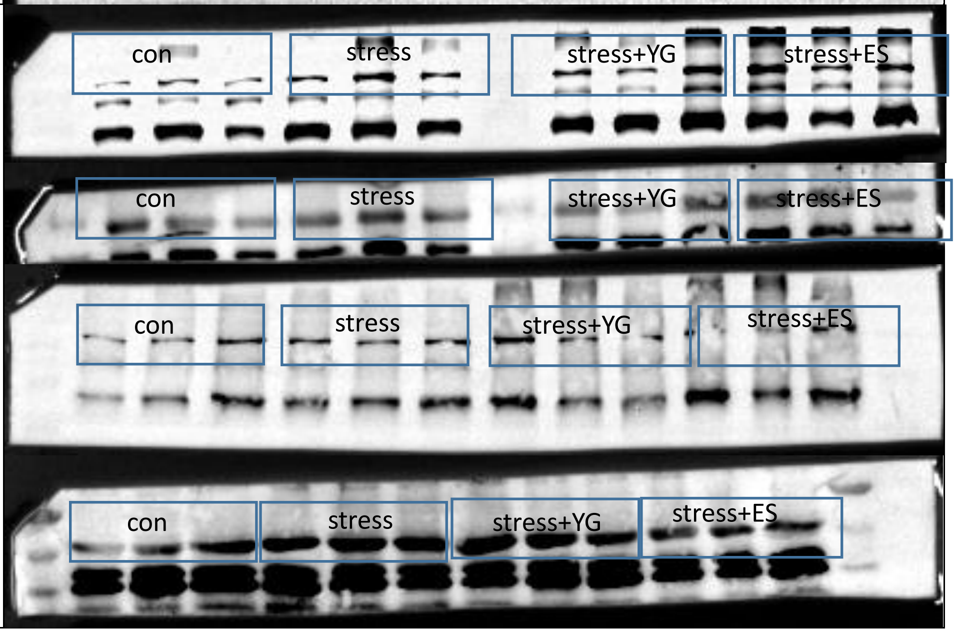

CamkII	Female	CamkII-1<br>Tubulin-1<br>CamkII-2<br>Tubulin-2	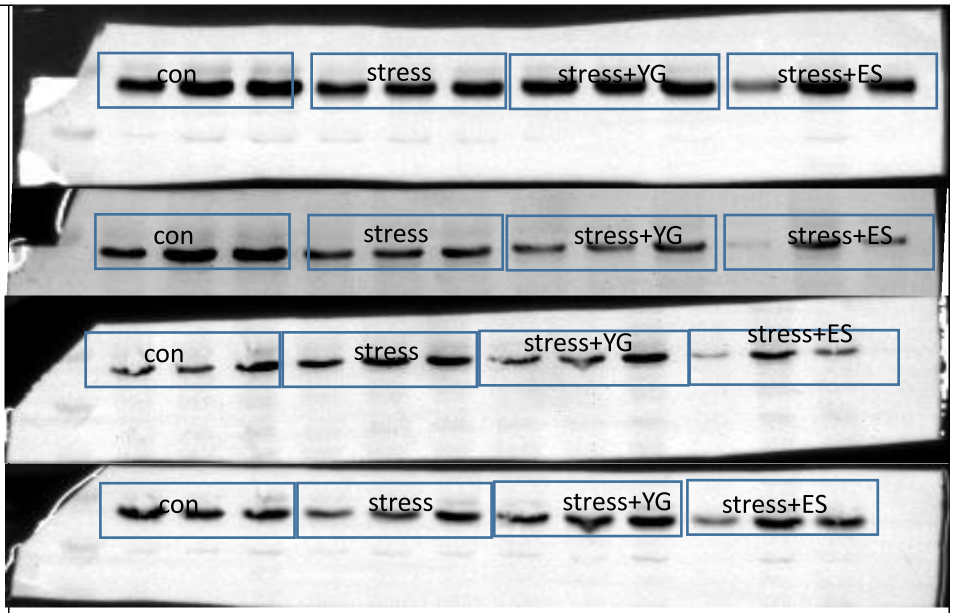
	Male	CamkII-1<br>Tubulin-1<br>CamkII-2<br>Tubulin-2	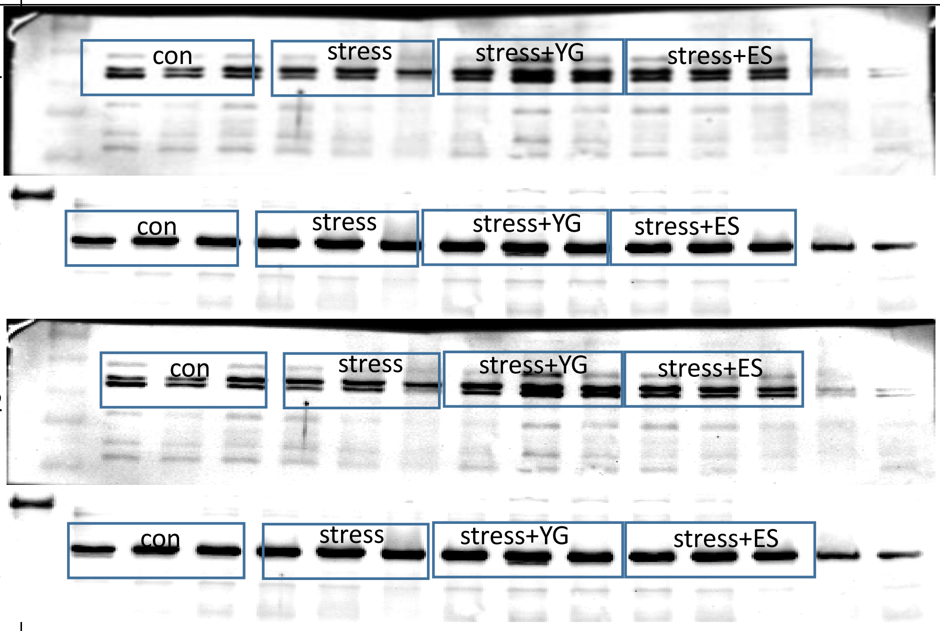
p-CREB	Con\stress	p-CREB-1<br>Tubulin-1<br>p-CREB-2<br>Tubulin-2	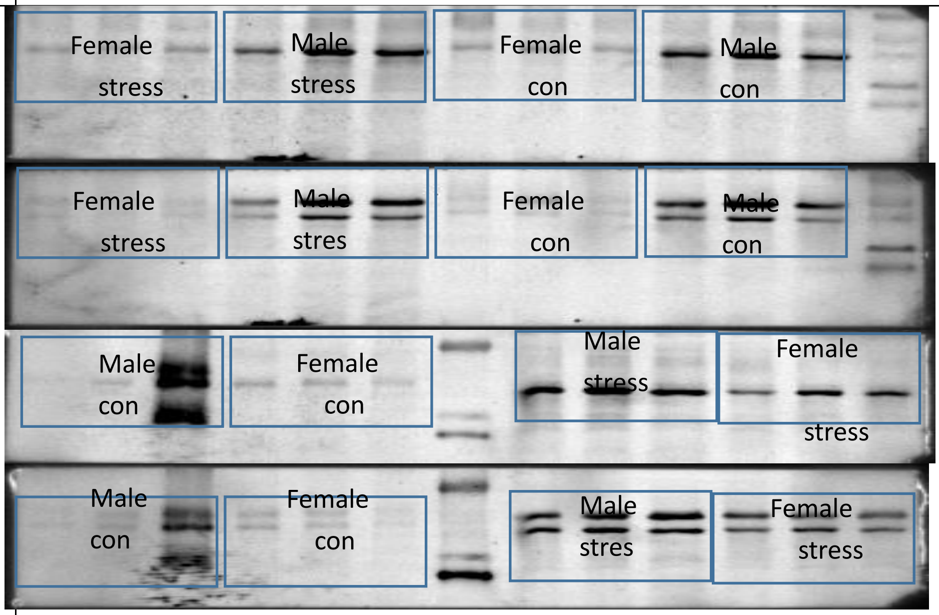

	stress+ YG/stress+ES	p-CREB-1<br>Tubulin-1<br>p-CREB-2<br>Tubulin-2	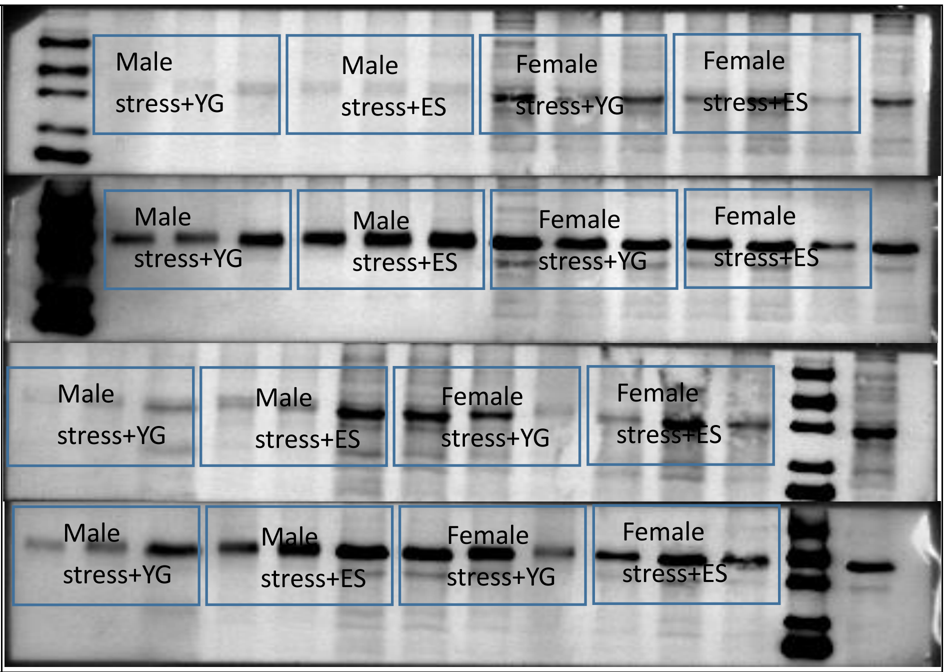
CREB	Con\stress	CREB-1<br>Tubulin-1<br>CREB-2<br>Tubulin-2	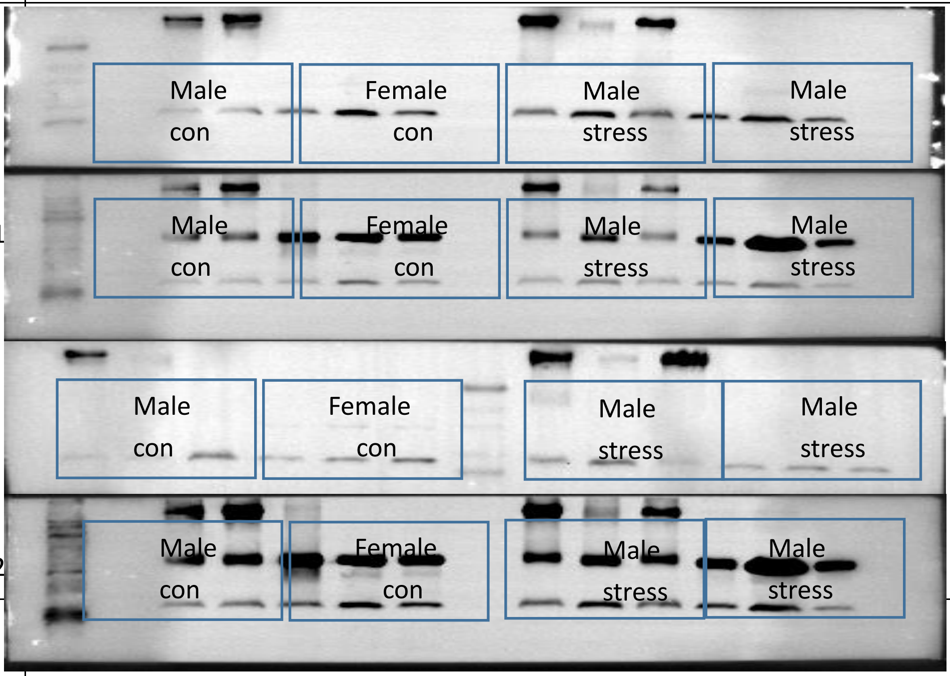
	stress+ YG/stress+ES	CREB-1<br>Tubulin-1<br>CREB-2<br>Tubulin-2	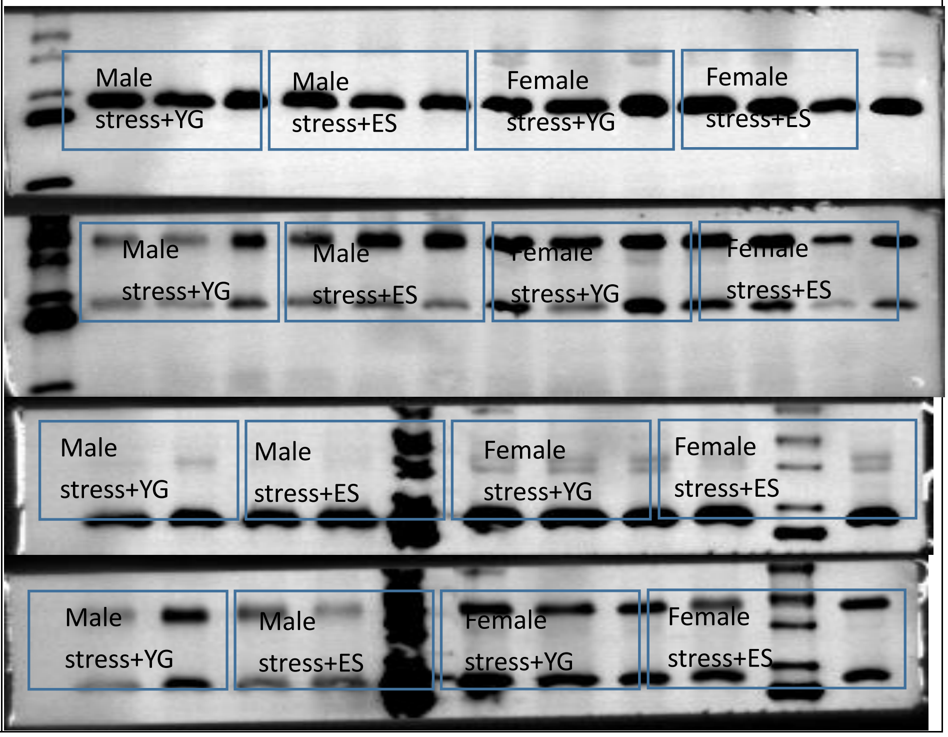

LPS	Female	nNOS1<br>CaMKII-1<br>GAPDH1<br><br>nNOS2<br>CaMKII-2<br>GAPDH2	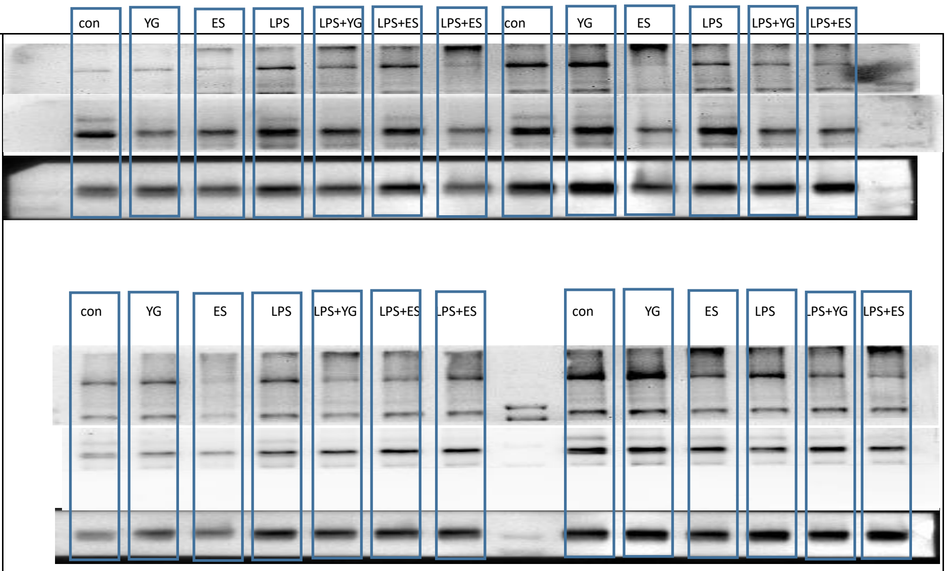
LPS	Male	nNOS1<br>CaMKII-1<br>GAPDH1<br><br>nNOS2<br>CaMKII-2<br>GAPDH2	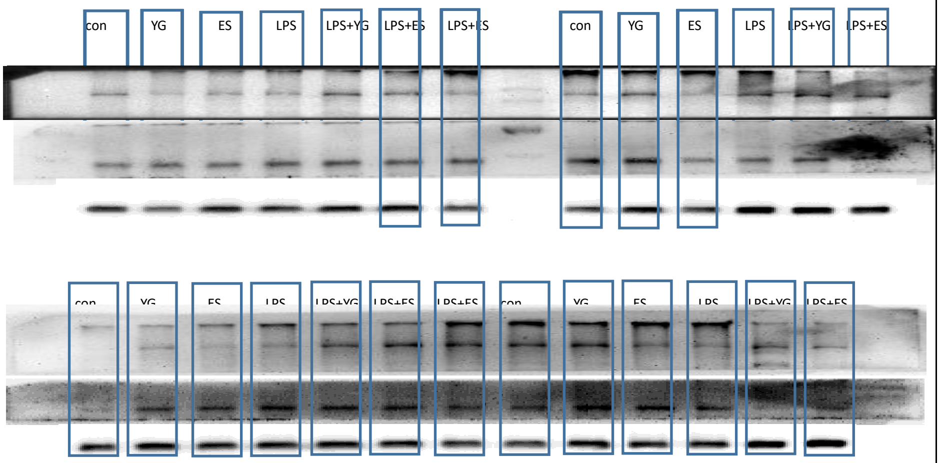
Baseline expression in female and male mice			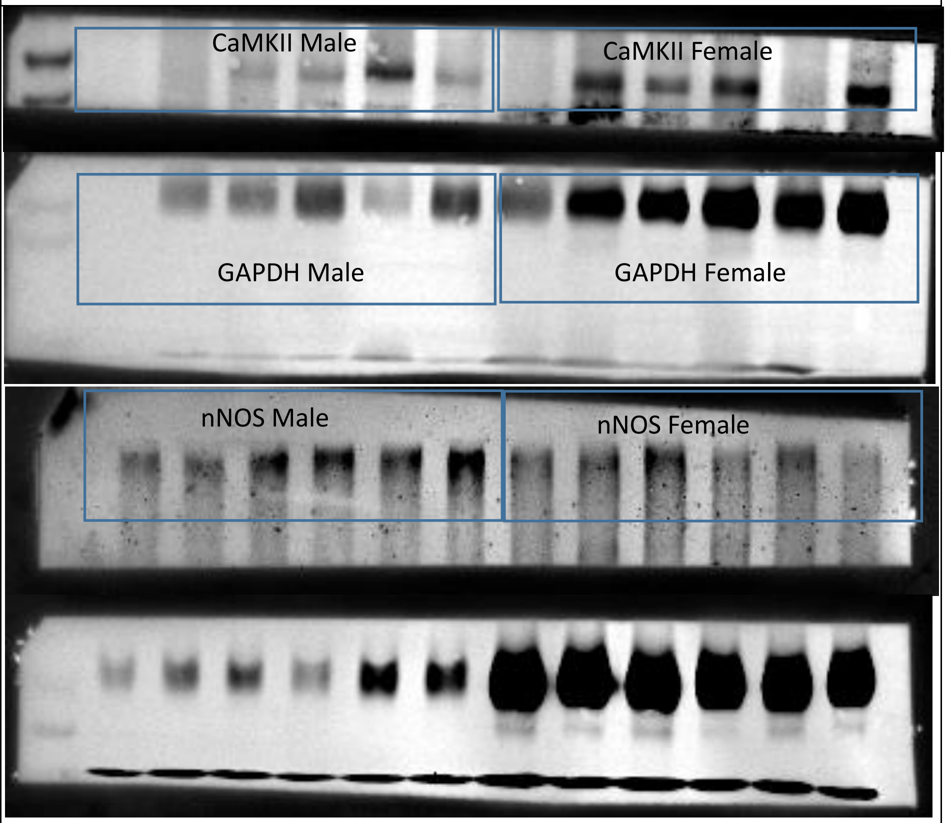
